# A non‐fluorescent immunohistochemistry method for measuring autophagy flux using MAP1LC3/LC3 and SQSTM1 as core markers

**DOI:** 10.1002/2211-5463.70014

**Published:** 2025-04-03

**Authors:** Shahla Shojaei, Amir Barzegar Behrooz, Marco Cordani, Mahmoud Aghaei, Negar Azarpira, Daniel J. Klionsky, Saeid Ghavami

**Affiliations:** ^1^ Department of Human Anatomy and Cell Science College of Medicine, University of Manitoba Winnipeg Canada; ^2^ College of Pharmacy Rady Faculty of Health Sciences, University of Manitoba Winnipeg Canada; ^3^ Department of Biochemistry and Molecular Biology Faculty of Biological Sciences, Complutense University Madrid Spain; ^4^ Instituto de Investigaciones Sanitarias San Carlos (IdISSC) Madrid Spain; ^5^ Department of Clinical Biochemistry School of Pharmacy and Pharmaceutical Sciences, Isfahan University of Medical Sciences Isfahan Iran; ^6^ Shiraz Institute for Stem Cell & Regenerative Medicine Shiraz University of Medical Sciences Shiraz Iran; ^7^ Life Sciences Institute and Department of Molecular, Cellular and Developmental Biology University of Michigan Ann Arbor MI USA; ^8^ Children Hospital Research Institute of Manitoba University of Manitoba Winnipeg Canada; ^9^ Research Institute of Oncology and Hematology, Cancer Care Manitoba University of Manitoba Winnipeg Canada; ^10^ Faculty of Medicine Academy of Silesia Katowice Poland

**Keywords:** autophagometer, autophagy flux measurement, cellular homeostasis analysis, chromogenic detection, cost‐effective autophagy assay, non‐fluorescent immunohistochemistry

## Abstract

Macroautophagy/autophagy is a crucial cellular process for degrading and recycling damaged proteins and organelles, playing a significant role in diseases such as cancer and neurodegeneration. Evaluating autophagy flux, which tracks autophagosome formation, maturation, and degradation, is essential for understanding disease mechanisms. Current fluorescence‐based methods are resource‐intensive, requiring advanced equipment and expertise, limiting their use in clinical laboratories. Here, we introduce a non‐fluorescent immunohistochemistry (IHC) method using MAP1LC3/LC3 and SQSTM1 as core markers for autophagy flux assessment. LC3 levels reflect autophagosome formation, whereas SQSTM1 degradation and a decrease in the number of its puncta indicate active flux (i.e., lysosomal turnover). We optimized chromogenic detection using diaminobenzidine (DAB) staining and developed a scoring system based on puncta number and the percentage of stained cells. This accessible, cost‐effective method enables reliable autophagy quantification using a standard light microscope, bridging the gap between experimental research and clinical diagnostics. Our protocol allows accurate autophagy evaluation in fixed tissues, offering practical applications in biomedical research and clinical pathology assessment.

## Graphical abstract

We introduce an immunohistochemistry method to measure autophagy flux, highlighting the active degradation and recycling of cellular waste. This cost‐effective approach uses tissue samples to track key markers like LC3 and SQSTM1, revealing how cells maintain health or respond to diseases such as cancer. It bridges the gap between research and clinical applications, offering practical diagnostic and therapeutic insights.

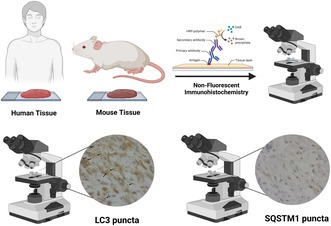

video

**Abbreviations**
ABC: avidin‐biotin complex
DAB: diaminobenzidine
DW: distilled water
IHC: immunohistochemistry
MAP1LC3B/LC3B: microtubule‐associated protein 1 light chain 3 beta
NaOH: sodium hydroxide
PBS: phosphate‐buffered saline
PMSF: phenylmethylsulfonyl fluoride
SDS: sodium dodecyl sulfate
SQSTM1: sequestosome 1

Autophagy is an essential cellular mechanism for degrading and recycling components, crucial for maintaining cellular homeostasis and responding to stress [1, 2]. This process is critical in various conditions, including cancer [3, 4], neurodegenerative diseases [5, 6], and infections [7, 8], highlighting its importance to health. Autophagy enables cells to dispose of damaged proteins and organelles, supporting renewal [9, 10]. Autophagy flux is a quantitative measure that assesses the dynamics of this process [11, 12]. Flux tracks the formation of autophagosomes, their fusion with lysosomes, and the degradation of their contents. This measure is vital for evaluating the efficiency and regulation of autophagy under different conditions or stimuli. Therefore, monitoring autophagy flux is crucial for understanding the roles of this pathway in biological and disease contexts [13]. However, traditional fluorescence‐based techniques have drastic limitations, prompting the development of alternative methods.

Although fluorescence‐based methods have been pivotal in advancing our understanding of autophagy, their utility is marred by significant drawbacks. These techniques require sophisticated equipment, specialized reagents, and a high level of technical expertise, escalating the costs and limiting their accessibility to laboratories having substantial resources [14, 15]. Furthermore, these methods prove impractical in clinical settings, where rapid and cost‐effective diagnostics are essential. Additionally, their application to fixed‐tissue samples frequently leads to complications such as tissue autofluorescence, which can mask accurate signals and necessitate extensive sample preparation [16]. This combination of high expense, technical complexity, and practical challenges in clinical environments accentuates the urgent need for more accessible and adaptable methods in autophagy research.

To address these limitations, we propose a novel non‐fluorescent immunohistochemistry (IHC) method for evaluating autophagy flux. This approach utilizes antigen–antibody reactions to detect and localize specific antigens, providing invaluable diagnostic, prognostic, and predictive information [17]. We exploit the specificity and robustness of immunohistochemical staining without relying on fluorescence. The specificity is crucial in distinguishing between different autophagic structures, which can overlap in fluorescent studies, while robustness ensures consistent, repeatable results across various sample types and conditions. Using chromogenic detection, this method provides clear and interpretable results that are easily quantifiable under a standard light microscope. The non‐fluorescent IHC method is cost‐effective and more straightforward than fluorescent approaches, making it accessible to a broader range of laboratories, including those in clinical settings.

This non‐fluorescent IHC method offers several significant advantages. First, it reduces costs associated with expensive fluorescent dyes and advanced imaging equipment, eliminating the need for specialized fluorescence microscopes and costly fluorochrome‐conjugated antibodies [18]. Second, it simplifies the technical requirements, making it feasible for use in routine clinical laboratories and research settings with limited resources [19]. Third, this method is highly applicable to patient samples, enabling fast and reliable assessment of autophagy flux in various clinical conditions [20]. These features facilitate timely and informed decision‐making in clinical diagnostics and research. With this IHC method, we can study autophagy mechanisms in diseases such as cancer and neurodegenerative disorders, where understanding cellular degradation processes is crucial.

This study aims to introduce the non‐fluorescent IHC method for evaluating autophagy flux and demonstrate its practical applications across clinical and research settings. By comparing this method with traditional fluorescence‐based techniques, we underscore its superior effectiveness, cost‐efficiency, and user‐friendliness. We anticipate that this innovative approach will transform the assessment of autophagy in patient samples, emerging as a crucial tool for clinical diagnostics and profoundly enhancing our understanding of autophagy‐related diseases.

Our innovative non‐fluorescent IHC method represents a significant advancement in autophagy research. By offering a cost‐effective, accessible, and reliable technique, this approach holds the potential to transform clinical diagnostics and research, making the evaluation of autophagy flux more practical and widespread.

## Material

### Buffers, diluent, and solutions

1. Citrate buffer: Mix 4.5 mL of 0.1 m citric acid (Sigma, 251 275‐500G) with 20.5 mL of 0.1 m sodium citrate (Millipore Sigma Canada Ltd., Oakville, ON, Canada, S‐4641‐500G), and add 225 mL of distilled water (DW) to reach the final volume of 250 mL.
2. Maleic acid buffer: Add 100 mm (116.1 g) maleic acid (Sigma, M0375‐500G) and 150 mm (87.5 g) NaCl (Fisher Scientific, Ottawa, ON, Canada, S271‐3) into an Erlenmeyer flask and add DW to approximately 800 mL. Adjust the solution to pH 7.5 using 40–70 g of NaOH. After adjusting the pH, increase the volume to 1 liter. Notes: (A) The maleic acid will only dissolve once the pH reaches 5.5–6. Once it dissolves, add the NaOH pellets very carefully to avoid overshooting the pH. (B) While adjusting the pH, ensure that the temperature is maintained between +15 and +25 °C as temperature variations can affect the pH.
3. Blocking stock solution (10×): Prepare a 10% Blocking Reagent (Roche, Mississauga, ON, Canada, 11 096 176 001) in the maleic acid buffer. Dissolve the blocking reagent with heating and shaking while avoiding boiling. Watch carefully to make sure the blocking reagent is completely dissolved. Autoclave and store at 2–8 °C; if the reagent is stored at −20 °C, it can be used for one year.
4. Blocking working solution (1×): Mix 0.5 mL of the blocking stock solution with 1.5 mL of maleic acid buffer, 0.5 mL fetal bovine serum/FBS (Thermo Fisher Scientific, Ottawa, ON, Canada, 26 140 079), 50 μL of 10% Tween‐20 (Thermo Fisher Scientific, BP337‐100) and 2.5 mL phosphate‐buffered saline (PBS; Milipore‐Sigma, 56 639). Use a vortex to completely mix.
5. Avidin‐biotin blocking solution (Vector Laboratories, Inc., Newark, CA, USA, SP‐2001): Ready to use.

### Substrates, chromogens, and counterstain solutions

1. Avidin‐Biotin Complex (ABC): ABC, an avidin‐biotin‐based enzymatic amplification system, has many biotinylated horseradish peroxidase/HRP molecules cross‐linked to avidin, with at least one biotin‐binding site able to bind to the secondary antibody and amplify the signal. Prepare ABC solution by adding 1 drop from bottle “A” (Vector, PL‐6100) and 1 drop from bottle “B” to 2.5 mL of PBS. Leave the solution for 30 min at room temperature to be activated. Note: This solution is stable for 1 week at 4 °C.
2. Peroxidase chromogen kit, diaminobenzidine (DAB): Prepare DAB substrate by adding 125 μL of 1% DAB (Thermo Fisher Scientific, 34 002) stock solution and 50 μL of 30% H_2_O_2_ to 2.5 mL PBS. Note: The substrate should be prepared fresh just before use. See Notes and Tips 1.
3. Counterstain: Add 10 drops of Mayer hematoxylin (Vector, H‐3404) to 1.25 mL of PBS.
4. Bluing reagent for counterstaining: Prepare 2% sodium bicarbonate (Thermo Fisher Scientific, BP328‐1), pH 8 in DW.

### Antibodies

1. Anti‐SQSTM1/p62 (D5L7G) primary antibody: Prepare a 1:50 dilution of SQSTM1/p62 (sequestosome 1) antibody (New England Biolabs, Ltd., Whitby, ON, Canada) in blocking working solution. Note: Primary antibody concentration may vary depending on the specific system being used. To optimize primary antibody concentration in your system, perform a serial diution around the recommended starting concentration. See Notes and Tips 2.
2. Anti‐MAP1LC3B/LC3B primary antibody: Prepare a 1:50 dilution of MAP1LC3B/LC3B (microtubule associated protein 1 light chain 3 beta)‐Specific Polyclonal antibody (ProteinTech Group, Inc. Rosemont, IL, USA, 18725‐1‐AP) in blocking working solution. Note: The primary antibody concentration may vary depending on the specific system being used. To optimize the primary antibody concentration in your system, perform a serial dilution around the recommended starting concentration.
3. Biotinylated anti‐mouse secondary antibody: IHC Select Secondary Goat Anti‐Mouse IgG Antibody, prediluted, biotinylated (Sigma‐Aldrich, 21538‐M).
4. Biotinylated anti‐rabbit secondary antibody: IHC Select Secondary Goat Anti‐Rabbit IgG Antibody, prediluted, biotinylated (Sigma‐Aldrich, 21537).

### Miscellaneous

1. Silanized slides: To prevent tissue detachment, use silanized (positively charged) slides (Sigma, S4651).
2. Mounting medium: To form a semi‐permanent seal for prolonged slide storage at 4 °C use Flourmount‐G (Invitrogen, Ottawa, ON, Canada, 00‐4958‐02).
3. Xylene, histological grade: For dehydration and rehydration steps, use ready‐to‐use xylene (Sigma‐Aldrich, 534 056).
4. Ethanol: For dehydration and rehydration steps, use food‐grade ethanol with high purity (UN 1170; Greenfield Global, Chatham, ON, Canada, PO25EAAN).

### Ethics

All tissues used in this study were obtained from commercial tissue microarray slides provided by US Biomax, ensuring compliance with ethical standards for the use of pre‐collected and commercially available samples.

## Method

Unless otherwise specified, perform all steps at room temperature.

### Day 1

1. De‐paraffinize and rehydrate slides. Carry out the following exchange at room temperature, manually in Coplin jars (Millipore‐Sigma, S5641), or using an automated embedding system. See Notes and Tips 3.
  - Warm the slides at 60 °C for 30 min in the Coplin jar.
  - Place the slides in xylene for 5 min (3×), being sure to cover the samples in this and all subsequent steps.
  - Place the slides in 100% ethanol for 1 min (2×).
  - Place the slides in 95% ethanol for 1 min.
  - Place the slides in 70% ethanol for 1 min.
  - Wash with running tap water for 1 min.
  - Place the slides in PBS for 1 min (3×).
2. Heat‐induced antigen retrieval:
  - Pre‐warm citrate buffer in a polypropylene Coplin jar. See Notes and Tips 4.
  - Place the slides in the Coplin jar.
  - Place the Coplin jar in a boiling water bath for 30 min.
  - Remove the Coplin jar from the boiling water.
  - Allow to cool slowly at room temperature for 20 min.
3. Blocking of endogenous proteins. Blocking steps are crucial in IHC to prevent excessive background staining in images. See notes and tips 5.
  - Remove slides from the citrate buffer one by one and dip quickly in double‐distilled H_2_O.
  - Gently blot on a paper tissue to remove liquid.
  - Circle the tissue sections with an ImmEdge pen (Vector, H‐4000).
  - Immediately cover the tissue sections with PBS. Note: Do not allow the sections to dry.
  - Apply PBS to the sections for 5 min to wash (3×).
  - Apply the blocking solution to the sections for 30 min.
  - Apply PBS to the sections for 5 min to wash (3×). Note: If you are using an antibody produced in the same species as your tissue (e.g., a mouse antibody on mouse tissue), blocking endogenous IgG is necessary. For example, use goat anti‐mouse IgG Fab fragment (Jackson ImmunoResearch, 115‐007‐003) diluted 1 : 10 in PBS for a minimum of 1 h. It is best to incubate overnight at 4 °C. Then, wash sections in PBS for 5 min (3×). If this is not an issue, proceed to the next step.
  - Apply freshly prepared 3% H_2_O_2_ (in PBS) for 10 min. This step is necessary to eliminate endogenous hydrogen peroxidases. Note: The incubation time may have to be increased to 15 or 20 min if the tissue has a lot of blood.
  - Apply PBS to the sections for 5 min to wash (3×).
  - Apply avidin blocking solution for 15 min. Note: This solution works just as well when diluted in an equal volume of PBS.
  - Apply PBS to the sections for 5 min to wash (3×).
  - Apply biotin‐blocking solution for 15 min. Note: This solution works just as well when diluted in an equal volume of PBS.
  - Apply PBS to the sections for 5 min to wash (3×).
4. Immunostaining:
  - Arrange sections in a moist chamber to avoid drying of tissues.
  - Apply primary antibody and incubate overnight at 4 °C. Note: Leave one section without primary antibody, and cover only with blocking solution to be used as a negative control. See Notes and Tips 7.

### Day 2

- Apply PBS to the sections for 5 min to wash (3×).
- Apply biotinylated secondary antibody (from the appropriate organism corresponding to the primary antibody) to all sections (including the negative control) for 30 min.
- Apply PBS to the sections for 5 min to wash (3×).
- Apply activated “ABC” solution for 30 min.
- Apply PBS to the sections for 5 min to wash (3×).
- Apply freshly prepared DAB substrate to the sections for up to 2 min. See Notes and Tips 8.
- Stop reaction by immersing slide in double‐distilled H_2_O in a Coplin jar.
- Wash slides with H_2_O in a Coplin jar (3×).
- Counterstain with Mayer hematoxylin for 1–4 min. See Notes and Tips 9.
- Wash slides with H_2_O in a Coplin jar (3×).
- Immerse in 2% sodium bicarbonate solution, pH 8 for 20 s.
- Wash slides with H_2_O in a Coplin jar (3×).

5 Dehydration and mounting. Dehydration is done by reversing the steps of hydration. Carry out the following exchange at room temperature, manually in Coplin jars, or using an automated embedding system. See Notes and Tips 10.
  - Place the slides in 70% ethanol for 1 min.
  - Place the slides in 95% ethanol for 1 min.
  - Place the slides in 100% ethanol for 1 min (2×).
  - Place the slides in xylene for 5 min (3×).
  - Remove slides one by one.
  - Blot the edge of the slide to a paper tissue to remove the extra liquid.
  - Add one drop of mounting medium on the top of the tissue section.
  - Mount a coverslip on the mounting medium.
  - Gently press the coverslip with the tip of a pencil to remove any possible bubbles.
  - Lightly clean the extra mounting agent surrounding the coverslip using the edges of a paper tissue.
  - Leave slides at room temperature overnight to ensure they are completely dry.

### Day 3

#### Imaging

To perform microscopy imaging for DAB IHC, tissue sections are prepared and stained with primary and secondary antibodies, followed by a DAB substrate, then mount the stained sections on slides and cover them with coverslips as described above. Use a bright‐field microscope equipped with a camera. Adjust the light and focus to visualize the brown DAB precipitate. Capture images at multiple magnifications (e.g., 10×, 20×, 40×). Ensure consistent imaging settings for all samples. Save and label images appropriately for analysis. This protocol provides clear visualization of DAB‐stained tissue sections, facilitating accurate evaluation and documentation.

### Day 4

#### Scoring

To ensure unbiased and accurate results, scoring DAB IHC for LC3B and SQSTM1 puncta involves a meticulous evaluation by three pathologists in a blinded manner. Each pathologist independently examines stained tissue sections under a microscope, focusing on the presence and intensity of the brown DAB residue, which indicates the presence of the target antigens LC3B and SQSTM1. The scoring process includes assessing the number of puncta staining and the percentage of positively stained cells. The puncta numbers for LC3B and SQSTM1 is graded on a scale from 0 to 3, where 0 indicates no puncta staining (null), 1 indicates low number of puncta staining (low), 2 indicates moderate numbers of puncta staining (medium), and 3 indicates high numbers of puncta staining (high). The percentage of positively stained cells is categorized into ranges (e.g., 0–25%, 26–50%, 51–75%, and 76–100%).

Each pathologist assigns scores independently, unaware of the others' assessments or identifying information about the samples. This blinded approach minimizes bias and enhances the reliability of the results. After scoring, the results from the three pathologists are compared and averaged to obtain a final score for each sample. This rigorous evaluation method ensures that the interpretation of LC3B and SQSTM1 puncta via DAB‐based IHC staining is accurate and reproducible, providing reliable data for research and diagnostic purposes. This process is critical for studies investigating autophagy, as LC3B and SQSTM1 are key markers of this pathway.

### Day 5

#### Autophagy flux evaluation, overview

Evaluating autophagy flux involves assessing the expression levels and patterns of cytosolic LC3B and SQSTM1 puncta, which are crucial markers of autophagy activity. Autophagy flux refers to the dynamic autophagosome formation, maturation, and degradation process. Note that there is an ongoing balance between LC3B and SQSTM1 synthesis and degradation. During autophagy induction, LC3B levels in particular increase, although the extent can be tissue‐ and cell‐type specific. LC3B is present on both sides of the phagophore, the initial sequestering compartment, and the autophagosome. The LC3B on the outer surface of the autophagosome is removed and recycled, whereas the population on the inner surface is exposed to the lysosome lumen and degraded, lowering the LC3B level. SQSTM1 is bound to LC3B on the inner surface of the phagophore and autophagosome and hence is also degraded in the lysosome. Therefore, an increase in LC3B can reflect (A) autophagy induction or (B) a block in autophagosome‐lysosome fusion or degradation within the lysosome. Accordingly, measuring LCB3 alone is not sufficient to assess autophagic flux. In general, an accumulation of LC3B along with SQSTM1 would indicate a block in flux. In contrast, an increase in LC3B accompanied by a decrease in SQSTM1 could indicate high autophagy activity (a large increase in LC3B due to autophagy induction along with turnover of both proteins). Thus, the balance between the levels of LC3B and SQSTM1 provides substantial information about flux.

#### Specific interpretation and evaluation of puncta

To ensure accurate evaluation of autophagy flux, we used a 40× objective, balancing resolution, and field coverage for clear visualization of LC3B and SQSTM1 puncta while preserving tissue sections for future use. Although a 63× oil‐immersion lens could enhance resolution, we avoided oil immersion due to its potential to damage coverslips, making tissue sections unsuitable for additional staining or long‐term storage. This consideration aligns with best practices in histological specimen preservation.

The autophagy puncta typically range from 0.5 to 1.5 μm in diameter, as reported in prior autophagy flux studies [21]. This size range is clearly detectable using our 40× objective, allowing us to distinguish autophagic structures while maintaining a broader field of view for representative tissue sampling. Puncta detection was qualitatively confirmed through multiple tissue sections and quantitatively assessed by counting at least 50 neural cell bodies per sample, following established autophagy monitoring guidelines [21].

**Fig. 1:**
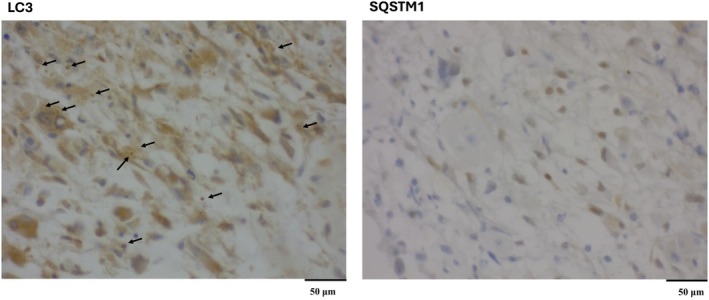
IHC analysis of autophagy flux in human brain tissue. Representative immunohistochemical staining of LC3B (A) and SQSTM1 (B) in tissue sections, indicating autophagy flux. LC3B staining shows high numbers of cytosolic puncta (score 3) (black arrow) with 75–100% positively stained cells, indicating high autophagosome formation. In contrast, SQSTM1 staining shows no cytosolic puncta staining (score 0) with 0–25% positively stained cells, reflecting the increase in LC3 puncta is due to active formation of autophagosomes. Based on these findings, the autophagy flux is categorized as “Very active autophagosome formation (II).” We used a 40× objective for taking the image.

To classify autophagy flux conditions, we evaluated both puncta size (≥0.5 μm) and the percentage of cells with cytoplasmic puncta, ensuring robust autophagy assessment. While SQSTM1 has known limitations due to additional regulatory mechanisms such as proteasomal degradation, combining it with LC3 remains a gold‐standard method for autophagy evaluation [21, 22]. This dual‐marker approach provided a reliable and reproducible method for assessing autophagic flux while minimizing sampling bias and maximizing specimen preservation (Fig. 1).

By carefully monitoring these patterns, researchers can infer the efficiency of the autophagic process, which is vital for understanding cell health and responses to various treatments or stressors. For a summary, please see Table 1.

**Table 1:** Autophagy flux indicators and interpretation.

IHC staining score of autophagy flux indicators		IHC staining interpretation
Cytosolic LC3B puncta	Cytosolic SQSTM1 puncta	
High^a^	High	Autophagy flux inhibited
High	Medium	Autophagy flux moderately inhibited
High	Low	Very active autophagosome formation (I)
High	Null	Very active autophagosome formation (II)
Medium	Null	^c^Very active flux (I)
Low^b^	Null	^c^Very active flux (II)
Low	Low	^c^Very active flux (III)
Null	Low	^c^Very active flux (IV)

^a^ High: 3; medium: 2; low: 1; null: 0

^b^ See Notes and Tips 6.

^c^ The cytosolic number of autophagosomes is determined by the balance between formation and degradation. If autophagosome formation increases without a proportional rise in degradation, they accumulate, reflecting impaired clearance. Conversely, efficient degradation prevents accumulation despite high formation. In the table, cdenotes classifications of very active flux states: I (medium formation, no degradation), II (low formation, no degradation), III (low formation, low degradation), and IV (no formation, low degradation). These classifications reflect different autophagic flux dynamics based on the interplay between autophagosome generation and clearance.

## Tips and tricks

Here are 10 important notes and tips for achieving optimal results with DAB IHC to measure autophagy flux:

1. Use fresh reagents: Ensure all reagents, especially DAB substrate, hydrogen peroxide, and antibodies, are fresh. DAB is light‐sensitive and can degrade, leading to reduced staining efficiency.
2. Optimize antibodies and concentrations: First, it is important to identify the appropriate antibodies that display optimal sensitivity, with the minimal amount of non‐specific binding and background. We tested several commercial antibodies and indicate in this article the ones we found to work best using our protocol. As new antibodies are likely to be developed, and current antibodies can vary depending on specific lot numbers, we recommend that researchers periodically test the available antibodies. Along these lines, we have chosen to use antibodies to LC3B and SQSTM1 in part because they are well characterized and widely available. However, depending on access, researchers and clinicians may decide to test additional antibodies directed against other Atg8‐family proteins such as those of the GABARAP subfamily, or against additional auto‐phagy receptors including NBR1, OPTN, and CALCOCO2. Second, finding the right dilution of primary and secondary antibodies is crucial for specific and strong staining. Use titration to determine the optimal antibody concentration for your specific tissue and antigen.
3. Proper tissue fixation: Over‐fixation or under‐fixation can adversely affect antigen availability. Standardize fixation time and conditions. Typically, 10% formalin for 24 h is effective for most tissues.
4. Antigen retrieval: Perform appropriate antigen retrieval methods, either heat‐induced (using a microwave, pressure cooker, or steamer) or enzymatic, to unmask epitopes. Use a retrieval buffer such as citrate or ethylenediaminetetraacetic acid/EDTA at the correct pH.
5. Control background staining: To minimize non‐specific staining, block endogenous peroxidase activity with hydrogen peroxide (usually 0.3% in methanol). Use normal serum or bovine serum albumin/BSA to block non‐specific binding sites.
6. Use appropriate controls: Always include positive and negative controls. These include a tissue known to express the antigen (positive control) and a sample in which the primary antibody is omitted or replaced with an irrelevant antibody (negative control). Low levels of LC3B may indicate efficient turnover of the protein, or a low level of autophagy induction. The extent of LC3B upregulation also varies depending on the tissue or cells being evaluated. Thus, in situations where there are low levels of both LC3B and SQSTM1 we recommend caution in interpretation of the results unless additional methods of evaluation are performed. For researchers using cell culture appropriate controls will include the addition of lysosomal protease inhibitors such as E64d and leupeptin with and without an MTOR inhibitor such as torin 1, which will induce autophagy. The combination of MTOR and protease inhibitors will indicate the highest levels of LC3B and SQSTM1 that can be obtained in the particular cell lines being used. See reference [21, 23] for additional information.
7. Optimized incubation times and temperatures: Incubation times and temperatures for primary and secondary antibodies should be optimized. Overnight incubation at 4°C for the primary antibody often enhances specificity.
8. Monitor DAB development time: The development time for DAB is critical. Overdevelopment can lead to non‐specific staining and high background. Monitor under a microscope and stop the reaction by washing with water as soon as the desired intensity is achieved.
9. Counterstaining: Use an appropriate counterstain (e.g., hematoxylin) to provide contrast and enable better visualization of tissue morphology alongside DAB staining. Optimize the counterstaining time to avoid obscuring DAB signals.
10. Proper mounting: Use aqueous or permanent mounting media compatible with DAB. Ensure that slides are properly dried and coverslipped to prevent fading and preserve the staining.

These tips can significantly improve the reliability and quality of DAB‐based IHC staining, ensuring clear and interpretable results.

## Conflict of interest

M.C. has received honoraria/consultation contracts from EQA Certificados and OCA Global. The other authors declare no conflict of interest.

## Author contributions

SS and MA: Methodology, preparation of draft’ ABB and MC: Figure preparation, preparation of draft; NA: pathological evaluation and scoring (leader of pathology team); DJK: evaluation of the results, final edit, revision; SG: innovation of method, designing the method, resources, supervision, finalizing the results, final edit, revision.

## Data Availability

All raw data related to pathological scoring are available upon request. The raw data for immunohistochemistry (IHC) can be accessed at https://doi.org/10.5061/dryad.brv15dvkn.
